# Motor imagery of hand actions: Decoding the content of motor imagery from brain activity in frontal and parietal motor areas

**DOI:** 10.1002/hbm.23015

**Published:** 2015-10-09

**Authors:** Sebastian Pilgramm, Benjamin de Haas, Fabian Helm, Karen Zentgraf, Rudolf Stark, Jörn Munzert, Britta Krüger

**Affiliations:** ^1^ Bender Institute of Neuroimaging, Justus Liebig University Giessen Germany; ^2^ Institute of Cognitive Neuroscience, University College London United Kingdom; ^3^ Experimental Psychology University College London United Kingdom; ^4^ Institute for Sports Science, Justus Liebig University Giessen Germany; ^5^ Institute of Sport and Exercise Sciences, University of Muenster Germany

**Keywords:** fMRI, MVPA, motor imagery, action mapping, motor cortex

## Abstract

How motor maps are organized while imagining actions is an intensely debated issue. It is particularly unclear whether motor imagery relies on action‐specific representations in premotor and posterior parietal cortices. This study tackled this issue by attempting to decode the content of motor imagery from spatial patterns of Blood Oxygen Level Dependent (BOLD) signals recorded in the frontoparietal motor imagery network. During fMRI‐scanning, 20 right‐handed volunteers worked on three experimental conditions and one baseline condition. In the experimental conditions, they had to imagine three different types of right‐hand actions: an aiming movement, an extension–flexion movement, and a squeezing movement. The identity of imagined actions was decoded from the spatial patterns of BOLD signals they evoked in premotor and posterior parietal cortices using multivoxel pattern analysis. Results showed that the content of motor imagery (i.e., the action type) could be decoded significantly above chance level from the spatial patterns of BOLD signals in both frontal (PMC, M1) and parietal areas (SPL, IPL, IPS). An exploratory searchlight analysis revealed significant clusters motor‐ and motor‐associated cortices, as well as in visual cortices. Hence, the data provide evidence that patterns of activity within premotor and posterior parietal cortex vary systematically with the specific type of hand action being imagined. *Hum Brain Mapp 37:81–93, 2016*. © **2015 The Authors. Human Brain Mapping Published by Wiley Periodicals, Inc.**

## INTRODUCTION

Over the last two decades, motor simulation phenomena have attracted a great deal of attention in the field of cognitive neuroscience. One pioneer in this discussion was Marc Jeannerod **[** 2001] who postulated a functional equivalence between imagining and executing an action in his simulation theory. This proposes that every action involves a covert stage, and that this covert state spans the goal of the action, the means to reach it, and its sensory consequences.

A prominent situation corresponding to these so‐called covert actions is the conscious simulation of one's own actions, that is, motor imagery (MI). MI is defined as an internal, conscious, and self‐intended rehearsal of movements from a first‐person perspective without any overt physical movement [Crammond, 1997; Decety and Jeannerod, 1996; Hanakawa et al., 2008; Jeannerod, 1994; see Munzert et al., 2009; Vogt et al., 2013, for reviews]. On a neural level, it has been proposed that MI is a simulation that uses the motor system as a substrate [Lange et al., 2006; Jeannerod 2001]. This has been supported by several neuroimaging studies showing that roughly the same brain areas are involved in both motor execution and MI [Decety et al., 1994; Deiber et al., 1996; Hanakawa et al., 2008; Lotze et al., 1999; Porro et al., 1996]. More precisely, this neural network is believed to be organized around the following motor and motor‐related regions: the supplementary motor area (SMA), the premotor cortex (PMC), the primary motor cortex (M1), posterior parietal regions such as the inferior (IPL) and the superior parietal lobe (SPL), the basal ganglia (BG), and the cerebellum [Guillot et al., 2008; Lotze et al., 1999; Munzert et al., 2009].

Whereas the brain mechanisms underlying covert stages of bodily actions are considered to be based on motor representations within the core and broader motor system, the actual organization of these motor maps within these areas remains controversial [Aziz‐Zadeh et al., 2006; Buccino et al., 2001; Ehrsson et al., 2003; Filimon et al., 2007; Hauk et al., 2004; Keysers and Gazzola, 2009; Stippich et al., 2002; Wheaton et al., 2004; Wolfensteller et al., 2007; see Fernandino and Iacoboni, 2010, for a review). For example, many studies have provided evidence for an effector‐specific somatotopic motor mapping during action simulation (action observation, motor imagery) within the premotor cortex (PMC), the primary motor cortex (M1), and posterior parietal regions (inferior parietal lobe: IPL; superior parietal lobe: SPL) (Buccino et al., 2001; Ehrsson et al., 2003; Jastorff et al., 2010; Sakreida et al., 2005; Stippich et al., 2002; Wheaton et al., 2004]. However, for example, Rijntjes et al. [1999] proposed an alternative form of action mapping, which is more effector‐independent. Their study demonstrated that signing one's name with the hand is associated with activation of the same premotor regions as signing one's name with the foot. This suggests that there might be an effector‐independent, invariant representation for specific actions rather than a clear somatotopic coding for actions in at least some parts of the motor system.

Thus, action simulation might depend on similar neural representations of action content [Zentgraf et al., 2011]. In a recent study, we examined how motor maps are organized during motor imagery and action observation. For action observation, we found action representations in the premotor and posterior parietal region when observing hand and foot actions that differed with respect to their action goals. For MI, in contrast, we found activation sites that passed the threshold only for aiming movements [Lorey et al., 2014]. However, despite this inconsistent finding for MI, it is still possible for MI to rely on action representations that, however, might not be detectable when considering univariate response levels alone.

Traditionally, fMRI data have been analyzed by looking for overall activity changes in brain regions in response to a stimulus or a cognitive task [Friston et al., 1995]. This form of data analysis does not consider more distributed changes of activation patterns within a given area, which can occur in the absence of overall amplitude modulations. Newer approaches such as multivariate decoding [Haxby et al., 2001; Haynes and Rees, 2005; Kamitani and Tong, 2005] allow the detection and identification of such distributed response patterns, and to link them to a given stimulus or a specific (planned, executed or—potentially—imagined) action. Previous studies have shown that this technique allows the decoding of intended and executed types of hand actions from parietal and frontal motor areas [Gallivan et al., 2011a,b,2013; Oosterhof et al., 2012a] as well as from lateral occipitotemporal cortex [Oosterhof et al., 2012b]. A study by Filimon et al. [2014] demonstrated that fine‐grained patterns of activity in these areas could be distinguished according to whether they were evoked by the execution, observation, or imagery of a reaching action. In addition, MI and motor execution evoked similar mean amplitudes in premotor and parietal regions, demonstrating the added value of decoding procedures. Furthermore, recently, Park et al. [2015] examined which motor regions have the greatest predictive validity for imagined and executed hand movements. They found that executed and imagined movements were best predicted from M1 and SMA, respectively. A study on stroke patients conducted by Rehme et al. [2014] even showed that MVPA analyses of resting‐state fMRI data allowed a significant classification of individual patients with respect to their motor impairment. The current study addresses the question whether different types of imagined hand actions can be decoded from spatial patterns of BOLD signals in motor and motor‐related cortices. In an fMRI experiment, subjects worked on three experimental conditions and 1 baseline condition. In the experimental conditions, they had to imagine three different right‐hand actions: an aiming movement, an extension–flexion movement, and a squeezing movement. These actions were adapted from a model proposed by Schubotz [2004] and Schubotz and Cramon 2003. Their so‐called HAPEM (Habitual Pragmatic Event Map) model defines three different task types: a spatial task resembling our aiming movement, an object‐related task resembling our squeezing movement, and a rhythmic task type resembling our extension–flexion movement. We then applied multivoxel pattern analysis (MVPA) to decode the identity of imagined actions based on the spatial patterns of the BOLD signals they evoked in motor, premotor and posterior parietal cortices. Separate multivariate classifiers were trained and tested for each region of interest (ROI) to obtain an index of pattern discriminability. We hypothesized that MI relies on different action‐dependent motor representations and that we would therefore be able to decode imagined action type above chance level within the frontal (e.g., M1, PMC) and posterior parietal motor areas (e.g., IPL, IPS, SPL). However, we expected best classification results in those areas associated with higher level aspects of movement planning, such as preparation and organization of movements in the premotor area [Wise, 1985], or coding of movement intention and decision making in the posterior parietal cortex [Andersen and Buneo, 2002; Desmurget et al., 2009].

## MATERIALS AND METHODS

### Subjects

Twenty right‐handed volunteers (12 females, mean age = 26.3 years, SD = 4.4) with normal or corrected‐to‐normal vision participated in this experiment. They reported no history of psychiatric or neurological disorders, and no history or current use of any psychoactive medication. The study was approved by the local ethics committee of the Psychology and Sport Science Department of the Justus Liebig University Giessen, and all subjects gave informed written consent in accordance with the Declaration of Helsinki. The study took place at the Bender Institute of Neuroimaging (BION, Justus Liebig University).

### Design and Task

The experiment consisted of three imagery conditions and one rest condition. Before the fMRI experimental phase, subjects completed a familiarization session (see below). In the MI conditions, they were instructed to imagine either a force production task squeezing a bellows, an aiming task pointing with the index finger at five targets affixed to the bellows, or an extension**–**flexion movement with the right hand (i.e., the fingers) alongside the bellows. The aiming task required no memorizing of a special sequence of the targets, because subjects were instructed to simply imagine pointing to five affixed targets one after another [Lorey et al., 2014]. Thus, in total, subjects were scanned during four conditions: (a) MI of a right‐hand squeezing task, (b) MI of a right‐hand aiming task, (c) MI of a right‐hand extension**–**flexion task, and (d) the rest condition. Subjects kept their eyes closed during all four conditions.

**Figure 1:**
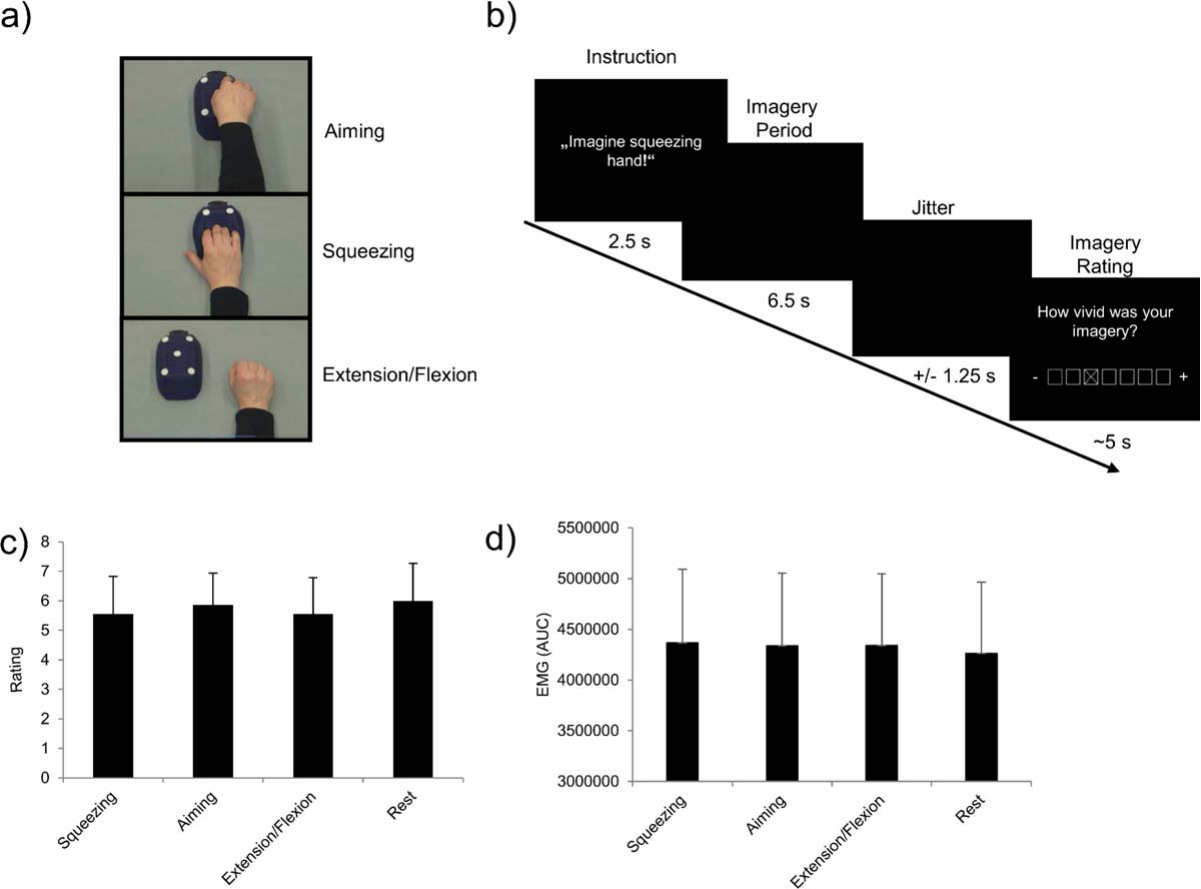
(**a**) Experimental conditions and (**b**) temporal structure of the experiment. (**c**) Subjective rating data: means and standard errors of the perceived imagery vividness. (**d**) EMG data: means and standard errors of the AUCs. [Color figure can be viewed in the online issue, which is available at http://wileyonlinelibrary.com.]

Conditions were presented in a pseudo‐randomized order counterbalanced across subjects. Each trial started with a written instruction presented for 2.5 s (*“Imagine Squeezing Hand, Imagine Aiming Hand, Imagine Rhythmic Movement Hand” or “Close Your Eyes and Rest”*), followed by a short delay (1 s) and the respective imagery or rest phase (6.5 s ± 1.25 s jitter; Fig. 1). Instructions were presented with a PC running Presentation software (Neurobehavioral Systems, Albany, USA) and projected onto a screen behind the scanner that could be viewed through a mirror attached to the head coil. During imagery and rest, subjects kept their eyes closed, reopening them only when the MI or rest phase was finished. This was signaled by a sound. Correct eye closure and opening were monitored with a video camera. After each trial, subjects were asked to rate the perceived quality (i.e., the perceived vividness) of their imagery performance on a 7‐point scale ranging from *very high* (7) to *very low* (1). Each subject performed 20 runs of eight trials each (corresponding to two trials in each of the four conditions) amounting to a total scanning time of approximately 50 min.

To control for involuntary movements during MI, we recorded the surface EMG sum potential from several target muscles of the right forearm during scanning (*M. extensor carpi radialis, M. extensor carpi ulnaris, M. flexor carpi radialis, M. flexor carpi ulnaris*).

### Familiarization Session

Prior to the fMRI experiment, subjects completed a preparatory session to familiarize themselves with the different experimental conditions and the experimental setting. First, they observed and executed the different actions (see above). Then, they imagined the actions, reporting the beginning and the end of each imagery phase by giving a sign with their left hand. This allowed the experimenter to check whether MI duration matched execution duration, thereby providing an estimate of compliance with the instructions. After each training trial, subjects rated the quality of imagery on a 7‐point scale ranging from *very high* (7) to *very low* (1). This session lasted a total of 20 min.

### Image Acquisition and Preprocessing

The fMRI data were collected on a 3 T whole‐body scanner (Siemens Prisma, Erlangen, Germany) with a standard 20‐channel head coil. We acquired not only a structural image from each participant consisting of 176 T1‐weighted sagittal images (1‐mm slice thickness; MPRAGE) but also a fieldmap (40 slices; TE (1): 10 ms; TE (2): 12.46 ms; TR: 1,000 ms).

For the run of functional imaging, a total of 1,000 volumes were registered using a T2*‐weighted gradient echo‐planar imaging sequence (EPI) with 40 slices covering the whole brain (slice thickness = 3 mm; 0.75 mm gap, descending; time of acquisition (TA) = 2.4375 s; time of repetition (TR) = 2.5 s; time of echo (TE) = 30 ms, flip angle = 87 degrees; field of view = 192 mm × 192 mm). The orientation of the axial slices was parallel to the AC–PC line. Trial onsets were jittered within a range of ±½ TR.

Image preprocessing was carried out using SPM8 (Wellcome Department of Imaging Neuroscience, University College London, UK). To find out whether head motion parameters in the scanner correlate substantially with the experimental conditions we calculated the maximum cosine between these parameters. For every subject we used the highest and thereby most unfavorable cosine. All cosine were below 0.3, and therefore the correlation was deemed not substantial. Origin coordinates were adjusted to the anterior commissure. Furthermore, mean bias correction, realignment, and unwarping were performed (using voxel displacement maps generated from the fieldmaps [Hutton et al., 2002] and the functional images were coregistered with the anatomical scan for the respective subject. Smoothing was executed with an isotropic three‐dimensional Gaussian filter with a full‐width‐at‐half‐maximum (FWHM) kernel of 5 mm.

### Data Analysis

#### Regions of interest

**Figure 2:**
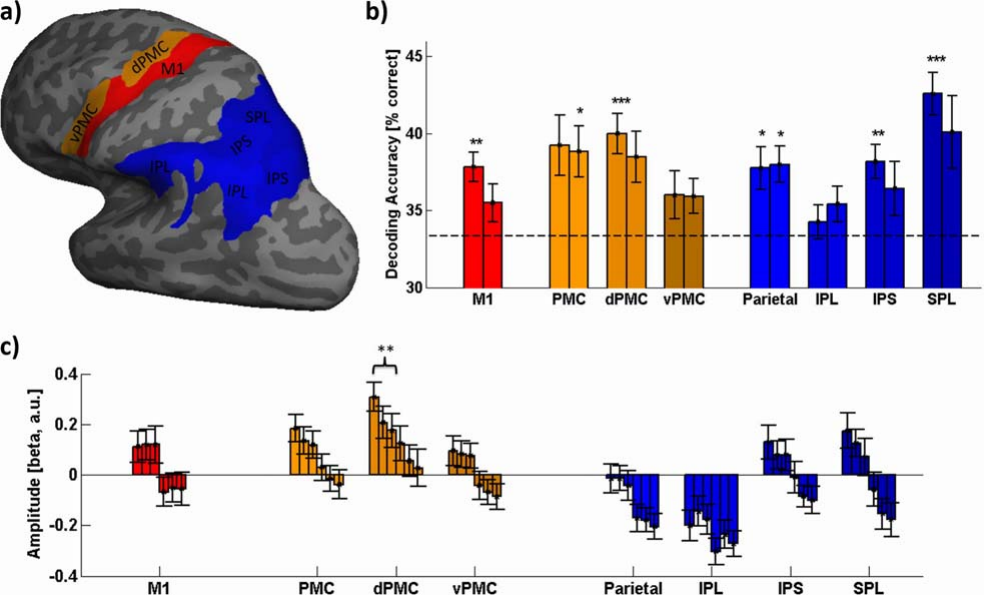
Regions of interest (ROIs), decoding results, and mean amplitude levels. (**a**) ROIs. The anatomical parcellation used for delineating ROIs is shown on the inflated left hemisphere of a representative participant. Labels refer to the ROIs shown in (**b**) and (**c**), which are given below. (**b**) Decoding results. Bars indicate the mean accuracy (% correct) with which the type of imagined action could be decoded from activation patterns in a given ROI. Left and right bars for each ROI represent the corresponding area of the left and right hemisphere, respectively. The dashed black line marks chance level. Asterisks indicate statistical significance of *t* tests versus chance level, adjusted for multiple testing using the Holm**–**Bonferroni method: *P < 0.05, **P < 0.01, ***P < 0.001. (**c**) Mean amplitude levels. Bars indicate the mean amplitude level across participants for a given ROI and condition (averaged across runs and voxels). The first three bars shown for each ROI (from left to right) correspond to: aiming, squeezing, and extension/flexion for the left hemisphere. Bars four to six for each ROI correspond to the same conditions in the same order for the right hemisphere. Asterisks indicate statistically significant differences in mean amplitude levels for left dPMC as determined with a repeated measures GLM: **p<.01, adjusted for multiple testing using the Holm**–**Bonferroni method. See Supporting Information for a “traditional” univariate analysis and activation map. M1: primary motor cortex, vPMC: ventral premotor cortex, dMPC: dorsal premotor cortex (with PMC as a combination of vPMC and dPMC), IPL: inferior parietal lobule, IPS: intraparietal sulcus, SPL: superior parietal lobule (with Parietal as a combination of IPL, IPS and SPL). All error bars indicate ± one standard error of the mean (S.E.M.). [Color figure can be viewed in the online issue, which is available at http://wileyonlinelibrary.com.]

The anatomical scan was used to reconstruct the cortical surface of each hemisphere using FreeSurfer (http://surfer.nmr.mgh.harvard.edu). Regions of interest (ROIs) were selected on the basis of previous findings reported in the MI literature (Ehrsson et al., 2003; Grèzes and Decety, 2001; Heed et al., 2011; Jeannerod, 2001] and defined anatomically on an individual basis using the FreeSurfer parcellation algorithm [Destrieux et al., 2010]. We defined eight ROIs per hemisphere as follows (cf. Fig. 2a):

- Primary *motor* cortex (M1), defined as the precentral gyrus
- Dorsal and ventral *premotor* cortex (dPMC and vPMC, defined as the superior and inferior part of the precentral sulcus, respectively) as well as a combined premotor region containing both parts of the precentral sulcus (PMC)
- Superior (SPL) and inferior *parietal* lobule (IPL, defined as the supramarginal and the angular gyrus, as well as the intraparietal sulcus (IPS), including transverse parietal sulci and a combination of these (Parietal)

Defining ROIs on an individual basis allowed us to work with high anatomical precision and avoided the need for spatial normalization. See Supporting Information Table S2 for details on ROI sizes.

#### General linear models

A first‐level analysis was computed with SPM 8 using separate general linear models (GLMs) for each subject and each of the 20 runs. We created four boxcar regressors corresponding to the four conditions. The boxcar functions of each regressor spanned the imagery or rest (for the rest condition) interval. Each regressor was convoluted with a canonical hemodynamic response function. Moreover, six movement parameters from the rigid‐body transformation of the motion‐correction procedure were entered as covariates in the GLM. The voxel‐based time series were filtered by a high‐pass filter (time constant = 128 s). Based on these GLMs, we calculated three contrast images per subject and run, each contrasting one of the MI conditions with the rest condition.

#### Multivariate pattern analysis

To test whether MI of different action types evoked separable response patterns in a given ROI, we conducted a linear discriminant analysis (LDA) with leave‐one‐run‐out cross‐validation for each subject using functions from the MATLAB statistics toolbox. We classified activations based on *t* contrast images derived from the GLM analysis described above [Misaki et al., 2010].

The *t* values within an ROI were vectorized for each contrast separately, deriving three response vectors per run. All vectors were subjected to a principal component analysis to reduce the number of features. Only the loadings of each voxel vector onto the first five principal components were entered into the decoding analysis. In each iteration of the cross validation, these shortened vectors were split into a set of test and training data corresponding to data from 1 and 19 runs, respectively. The LDA algorithm was provided with labels indicating the condition for each of the training examples and a linear decision hyperplane was derived on the basis of these data. This decision criterion was applied, in turn, to the test data and used to assign condition labels to each of the three test vectors.

We compared each of the assigned labels with the veridical labels and counted correct and incorrect assignments as 1 and 0, respectively. The whole procedure was repeated until each run had served as test data once, and we then calculated the proportion of correct assignments across the folds of this cross‐validation procedure. This proportion of correct assignments was derived separately for each subject and ROI and its difference from chance level (1/3) was tested across subjects using *t* tests. All *P* values were corrected for multiple ROIs using the Holm–Bonferroni method [Holm, 1979].

**Figure 3:**
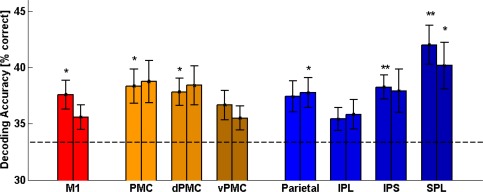
Decoding results for mean‐centered patterns. Bars indicate the mean accuracy (% correct) with which the type of imagined action could be decoded from mean‐centered activation patterns in a given ROI. Left and right bars for each ROI represent the corresponding area of the left and right hemisphere, respectively. The dashed black line marks chance level. M1: primary motor cortex, vPMC: ventral premotor cortex, dMPC: dorsal premotor cortex (with PMC as a combination of vPMC and dPMC), IPL: inferior parietal lobule, IPS: intraparietal sulcus, SPL: superior parietal lobule (with parietal as a combination of IPL, IPS, and SPL). Error bars indicate ± one standard error of the mean (SEM). Asterisks indicate statistical significance of *t* tests versus chance level, adjusted for multiple testing using the Holm**–**Bonferroni method: *P < .05, **P < .01, ***P < .001. [Color figure can be viewed in the online issue, which is available at http://wileyonlinelibrary.com.]

To test to which degree decoding performance depended on potential differences in mean amplitude results, we additionally mean‐centered all *t*‐maps and re‐ran the decoding analysis on these standardized patterns (Fig. 3).

#### Searchlight analysis

To test whether and where patterns of neural activity carried information about the content of motor imagery outside our ROIs, we ran an additional, exploratory searchlight analysis [Kriegeskorte et al., 2006; cf. de Haas et al., 2013]. For this analysis, we derived activation patterns from the same (trial‐specific and mean‐centered) *t*‐maps that we used for the ROI analysis described above. The searchlight consisted of a sphere with a radius of five voxels that was centered on each cortical grey matter voxel for each participant's brain in turn (using FreeSurfer segmentations excluding the cerebellum). For each iteration, the analysis was restricted to the grey matter voxels intersecting the respective searchlight sphere. The corresponding patterns were read out for each trial and we applied the same classification procedure as described for the ROI analysis. Classification accuracies were projected back onto the seed voxel, resulting in an accuracy map for each participant. We subtracted chance level (1/3) from these accuracy maps, spatially smoothed them with a small Gaussian kernel (FWHM 1 mm), normalized them to MNI space (http://www.loni.ucla.edu/ICBM/) and tested for whole brain family‐wise error (FWE) corrected significance at cluster level in SPM 8 (*P <* 0.05 FWE; voxel‐wise cluster forming threshold *P <* 0.001 uncorrected). Significant clusters were identified anatomically using the Juelich Histological [Eickhoff et al., 2005] Atlas implemented in the SPM Anatomy Toolbox (v. 2.0, http://www.fz-juelich.de/inm/inm1/DE/Forschung/_docs/SPMAnatomyToolbox/SPMAnatomyToolbox_node.html).

### Subjective Rating and EMG Data Acquisition and Analysis

After each trial in the fMRI session, subjects rated the success of each experimental trial on a 7‐point Likert scale ranging from *very high* (7) to *very low* (1). We calculated mean rating scores for each experimental condition, and computed an ANOVA to examine the effects of the respective action (aiming, squeezing, rhythmic extension**–**flexion) on the subjective ratings.

We analyzed EMG data collected in the fMRI session by determining the area under the curve (AUC) (duration of the averaged epoch: 5 s). These data were then averaged for each subject in each condition. The averaged data were subjected to multiple paired *t* tests comparing EMG activity for each imagery condition with EMG activity in the rest condition. All *P* values were corrected for multiple ROIs using the Holm**–**Bonferroni method [Holm, 1979].

## *R*ESULTS

### Subjective Ratings

All subjects gave high ratings in all experimental conditions (mean ratings >5.5). Conditions did not differ significantly from each other. All means and standard errors are depicted in Figure 1c.

### EMG Data

Muscular activity during MI was controlled during scanning. Multiple pairwise *t* tests for each imagery condition revealed no significant differences compared to resting baseline (Holm**–**Bonferroni adjusted). All means and standard errors are depicted in Figure 1d.

### Neuroimaging Data

#### Multivariate fMRI results: ROIs

To test whether response patterns in a given ROI carried information about the type of action imagined, we compared decoding performance in each ROI against chance level (cf. Fig. 2b). The imagined type of action could be classified significantly above chance level in left M1 (*t*_19_ = 4.72, *P =* 0.002), right PMC (*t*_19_ = 3.34, *P =* 0.04), left dPMC (*t*_19_ = 5.12, *P <* 0.001), left (*t*_19_ = 3.18, *P <* 0.05) and right Parietal (*t*_19_ = 3.95, *P =* 0.01), left IPS (*t*_19_ = 4.34, *P =* 0.004) and left SPL (*t*_19_ = 6.60, *P <* 0.0001).

To test how far these decoding results depended on the observed differences in mean amplitude results, we mean‐centered all *t* maps and reran the decoding analysis on these standardized patterns (Fig. 3). The imagined type of action could be classified significantly above chance level from mean‐centered patterns in left M1 (*t*_19_ = 3.33, *P* < 0.05), left PMC (*t*_19_ = 3.31, *P <* 0.05), left dPMC (*t*_19_ = 3.70, *P <* 0.05), right Parietal (*t*_19_ = 3.33, *P <* 0.05), left IPS (*t*_19_ = 4.59, *P <* 0.01), and left (*t*_19_ = 5.00, *P <* 0.01) and right (*t*_19_ = 3.29, *P <* 0.05) SPL. Because most of our ROIs showed the hypothesized effect, we tested the specificity of our results by trying to decode imagined hand actions from a control area that we expected to carry no such information: Heschl's gyri (as determined via the FreeSurfer parcellation). This control analysis confirmed that neither left (*t*_19_ = 0.98*, P =* 0.34), nor right (*t*_19_ = 0.46, *P =* 0.65), nor bilateral (*t*_19_ = 1.56, *P =* 0.13) Heschl's Gyri allowed significant decoding of imagined hand actions above chance level. All *P* values were corrected for multiple testing using the Holm**–**Bonferroni method. Significant decoding accuracies ranged from 5% (left M1) to 9% (left SPL) above chance level and were thus comparable to previous results for decoding intended and executed hand actions from evoked BOLD activations in (pre)motor and parietal areas [Gallivan et al., 2011b, 2013].

#### Searchlight results

**Figure 4:**
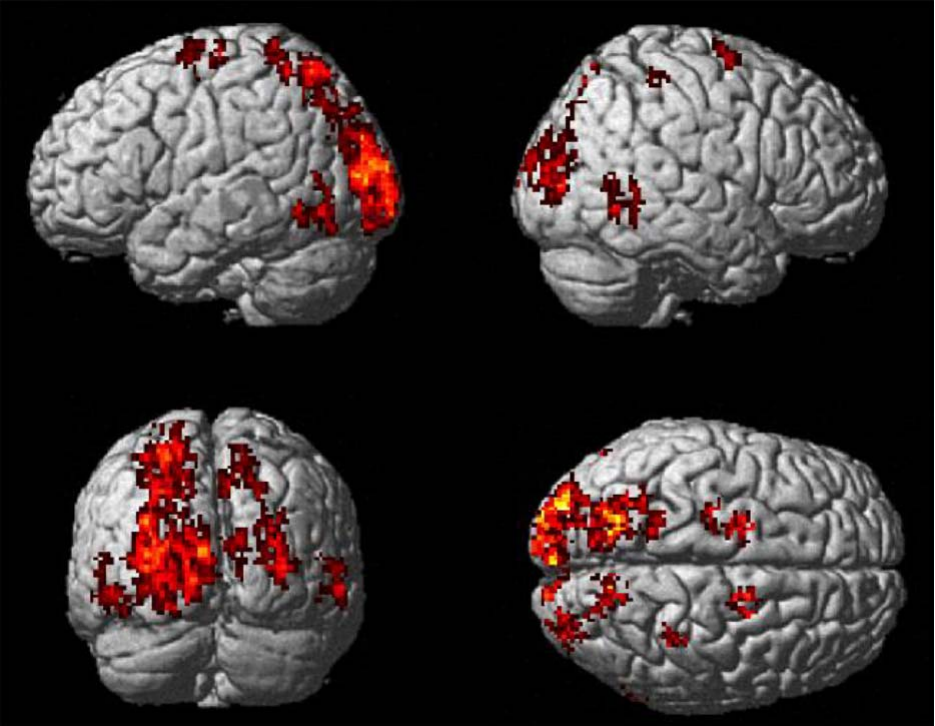
Results of the searchlight analysis. Heatmap colors indicate searchlight decoding performance for the respective seed voxels at group level (all *t* > 3.57; only significant clusters shown (*P <* 0.05 whole brain FWE‐corrected)). [Color figure can be viewed in the online issue, which is available at http://wileyonlinelibrary.com.]

In addition to our ROI analyses we used a searchlight approach to explore which regions of the brain carried information about imagined types of hand action (see Methods, above). This analysis confirmed that imagined hand actions could be decoded from activity patterns in left M1 and PMC, as well as right SPL (with one significant cluster in the left precentral gyrus, two in the left SMA, and one in right SPL). In addition, the searchlight analysis revealed significant clusters in right motor cortices (two stretching across pre‐ and postcentral gyrus, one in the SMA) and bilateral early visual cortex (EVC; two in the right hemisphere and one large cluster stretching from left EVC to left SPL), as well as bilateral human motion complex (V5/hMT+) and/or the extrastriate body area (EBA; left and right cluster stretching into fusiform gyrus and IPL, respectively; left peak coordinates being very close to EBA coordinates reported by Downing et al. [2001]). See Figure 4 and Table 1 for a summary and peak coordinates of all clusters.

**Table 1:** Searchlight results

			MNI coordinates of max. *t* value			
	Left/right	Cluster size	*x*	*y*	*z*	Max*. t* value
EVC/SPL	L	2,133	−32	−84	12	7.28
EVC	R	285	34	−82	4	6.98
EVC	R	92	14	−98	18	5.89
SPL	R	208	14	−64	62	5.51
hMT+/EBA	L	161	−54	−72	0	5.70
hMT+/IPL	R	132	60	−58	−6	6.05
BA6 (SMA)	L	116	−16	−4	72	4.80
BA6 (SMA)	L	37	−10	−6	56	4.79
BA6 (SMA)	R	113	12	−4	54	6.02
BA4 (M1)	L	61	−26	−20	68	5.54
BA4/2 (M1/S1)	R	47	34	−32	58	4.60
BA2/4 (S1/M1)	R	48	36	−34	46	5.65

Clusters with above chance decoding of imagery condition.

*P <* 0.05, whole‐brain FWE‐corrected on cluster level. For abbreviations, see text.

## DISCUSSION

The present study sought to address the question whether MI is accompanied by action‐dependent patterns of activation within the premotor, primary motor, and posterior parietal cortices. We used fMRI and MVPA to distinguish different hand‐related types of action (aiming, extension**–**flexion, squeezing) based on the fine‐grained patterns of BOLD activation they evoked in these areas. Our findings show that decoding of MI content is possible for the left premotor region, especially its dorsal section, the posterior parietal region of both hemispheres, the left intraparietal sulcus, the right inferior parietal lobe, the superior parietal lobe of both hemispheres, and the left M1. An exploratory searchlight analysis confirmed these results and extended them to bilateral motor and visual cortex.

Our data provide evidence that patterns of activity within motor, premotor, and posterior parietal as well as visual cortex differentiate between three different types of imagined hand actions: a force production task, an aiming task, or an extension**–**flexion task. Therefore, they suggest that frontal as well as parietal motor‐related areas represent the *content* of motor imagery. The core novelty of our results is that the evoked patterns of activity vary systematically, according to *which type* of action was imagined using the same effector. By training separate multivariate classifiers, we were able to decode the identity of three imagined hand actions that differ only with respect to their task type (spatial, object‐related, rhythmic) not with respect to the used effector. This complements and extents previous research showing that, first, patterns of activation in these areas differentiate *whether* a given type of action was imagined, observed, or executed [Filimon et al., 2014] and, second, *whether* a larger movement (wrist movement in hand rotation) or a smaller movement (finger movement in hand grasping) was imagined or executed [Park et al., 2015]. The following section will discuss these findings and their possible implications in more detail.

### Action‐Dependent Information Within a Distributed Network Over the PMC and the PPC

In a previous univariate fMRI study conducted in our lab [Lorey et al., 2014], we found action‐specific activation sites in the premotor and posterior parietal region when observing hand and foot actions that differed with respect to their action goals. For MI, in contrast, this previous study revealed action‐specific activation sites that passed the threshold only for aiming movements. Thus, these earlier results suggested broad overlapping activations for imagining different hand actions [Lorey et al., 2014]. In contrast, in the present study applying MVPA allowed us to identify areas for which voxel patterns of activation differentiate between the imagined types of hand actions, even when overall levels of signal amplitude across voxels of these areas did not differ. Thus, MVPA revealed separate representations of imagined action types, that were indistinguishable on a univariate level. The MVPA results demonstrate that information discriminating between different imagined types of hand action could be recovered from activation patterns in individual areas that collectively form a spatially distributed network.

This network spans from premotor and primary motor areas to the posterior parietal cortex. Within the frontal motor areas, discriminative information was carried by activation patterns in the premotor cortex, especially its dorsal section, as well as in M1. Regarding posterior parietal sites, patterns within SPL and within the IPS carried discriminative information. The highest accuracies were achieved for decoding patterns from left superior parietal lobe (note, however, that the validity of comparisons between areas might be limited, e.g., because of the different number of voxels contributing to the respective patterns).

In summary, action‐type‐related information could be retrieved from imagery‐evoked patterns within multiple regions of the core and broader motor network in frontal and parietal cortices. This suggests that information allowing the distinction between different imagined hand actions is represented over multiple frontal and parietal regions. Crucially, this information could be retrieved from activation patterns within each of these areas individually, which suggests that multiple imagined actions were represented in each area. This contradicts conclusions derived from univariate fMRI studies that argue in favor of a localized representation of specific types of actions [Lorey et al., 2014; Schubotz, 2007]. This difference might be due to a higher sensitivity of multivariate compared to univariate methods. An area representing a relevant dimension of imagined hand actions does not necessarily show a general increase in the level of activity when imagining the respective type of action. Rather, the corresponding information might be represented by differences in the fine‐grained pattern of activation that are lost in univariate analyses.

Both premotor and parietal areas have been discussed as being functionally organized with respect to motor acts and specific action goals. Some have argued that the cortical motor system has evolved primarily for the organization of different types of actions rather than for movement control per se [see Gallese et al., 1996; Kohler et al., 2002; Rizzolatti et al., 1988, for animal studies and Fernandino and Iacoboni, 2010, for a review]. In humans, Rijntjes et al. [1999] have identified action‐dependent activation maps with clusters corresponding to different types of action consequences. A similar principle of functional meaning has been discussed for posterior parietal areas indicating that motor acts with different goals activate segregated anatomical regions. For example, the observation of motor acts with similar goals activated the same anatomical locations regardless of the effector performing them. Similarly, Heed et al. [2011] have inferred that posterior parietal regions follow action‐centered organization principles. A former study by our own group demonstrated that activation within the premotor and the posterior parietal cortex differs according to action demands when observing hand and foot actions [Lorey et al., 2014]. These previous findings led us to speculate that premotor and parietal areas might contain an action‐based representation of motor acts for action simulation.

The present findings extend this view by showing that patterns of activation within motor, premotor, and parietal areas are distinct and specific to the respective imagery content. They, suggest that brain activations generated by MI of different types of actions differ in their fine‐grained, within‐area patterns of activation. Thus, activity in a single area appears to be involved in the representation—and possibly differentiation—of multiple types of actions.

### Action‐Dependent Information in Visual Cortex

An exploratory searchlight analysis revealed additional information carrying clusters in bilateral lateral occipitotemporal cortex (LOTC) as well as early visual cortex (EVC; Fig. 4; Table I). LOTC clusters probably intersected the human motion complex (V5/hMT+) and/or extrastriate body area [EBA; Downing et al., 2001; particularly its hand representation; Orlov et al., 2010]. This is in line with previous results, showing LOTC to carry information about *observed* manual actions [Oosterhof et al., 2012b] and extends them to motor imagery.

Imagined manual actions could also be decoded from early visual cortex, with clusters overlapping calcarine sulci in both hemispheres. This echoes recent results, showing that BOLD patterns in EVC carry information about purely auditory stimuli, as well as imagined auditory stimuli [Vetter et al., 2014]. A finding that in turn relates to evidence for crossmodal modulations of information content in visual cortex [de Haas et al., 2013] and has been interpreted to reflect feedback of predictive information to EVC [Friston, 2010; Vetter et al., 2014]. Likewise, our current finding of LOTC and EVC carrying information about imagined types of hand actions could be related to predictive feedback from parietal and motor areas. Alternatively, it could point to visual imagery of the first person perspective as part of the motor imagery employed by our participants.

Our searchlight uncovered a significant cluster in contralateral M1 that was situated just medially of the hand knob [Yousri et al., 1997]. A possible explanation for this is that seed voxels medially of the hand knob stand for searchlight spheres which covered the hand and wrist areas laterally as well as arm and trunk representations more medially. This might well be advantageous compared to a placement of the sphere directly on the hand knob, which would likely cover less of the arm and trunk representations medially, but extend to face representations laterally. Another possible interpretation is that the apparent medial shift is simply due to a lack of spatial precision introduced by the combination of spatial normalization [Ardekani et al., 2005], cluster‐level correction [Woo et al., 2014] and the size of the searchlight sphere.

### Implications and Future Directions

Based on the present MVPA results, we can conclude that neural activity within frontal and parietal motor regions can be informative for decoding the content of motor imagery, that is, of actions that differ with respect to action characteristics and action goals such as force production, precise aiming, or intransitive rhythm production performed with the same limb. At first glance, these findings might be taken to indicate that frontal and parietal areas represent imagined actions in a redundant manner. However, the present results do not tell us which dimension or part of the motor act actually is represented by frontal and/or parietal areas. Hence, these might differ between different areas, reflecting their different roles in motor behavior (see below).

It is well known that frontal and parietal motor areas are strongly interconnected and interact for many aspects of action planning. Regarding the role of the frontal lobe in action, it is widely accepted that the primary motor cortex (M1) is a source of specific motor commands [Evarts and Thach, 1969; Penfield and Boldrey, 1937], whereas more anterior regions of the frontal lobe such as the premotor region are involved in many higher level aspects of movement planning such as the preparation and organization of movements and actions [Wise, 1985]. Here, it has to be noted that especially the dorsal section of the premotor region is crucial for deciding which action to perform. The ventral premotor region, on the other hand, is discussed as being relevant for transforming target information in space into the motor information required for reaching, thus matching the visual to motor space [Hoshi and Tanji, 2007]. Posterior parietal cortex (PPC) has been considered to be important for movement intention and decision making [Andersen, 1987; Andersen and Buneo, 2002; Desmurget et al., 2009; Graziano and Gross, 1998; Kalaska et al., 1997; Rizzolatti et al., 1997], and a broad body of literature relates the process of state estimation to the posterior parietal cortex [Desmurget et al., 2009].

Alongside the general finding that MI content can be decoded from activity patterns within both frontal and parietal motor regions, our results also provide hints regarding differential roles of the various frontal and parietal subareas in representing action states and action characteristics (but see above for a caveat regarding the interpretation of interarea differences in decoding performance). For example, our findings show that a decoding of MI content is possible for the dorsal but not for the ventral premotor region. Thus, especially the activity within the section of the premotor area that is assumed to decide which kind of action is to be performed [Hoshi and Tanji, 2007] offers useful information for decoding action‐specific imagery content. The matching of visual to motor space on the other hand (ascribed to ventral premotor cortex) seems less informative regarding the type of imagined action. This might point to a less prominent role of this type of matching for motor imagery in general. Alternatively, it could be related to the specific types of actions we studied—all of the hand actions our participants imagined were confined to a small spatial region in front of them. Future studies could probe whether ventral premotor cortex allows us to distinguish between types of actions that vary more saliently along spatial dimensions.

A further discriminating result refers to the primary motor cortex. Here, our findings show that decoding of imagery content is possible only for the contralateral M1, but not for the ipsilateral. The fundamental function of the contralateral primary motor cortex is to control voluntary movements [cf. Sanes and Donoghue, 2000]. M1 neurons encode movement variables that have been termed higher order [Georgopoulos et al., 1982], for example, movement direction [Georgopoulos et al., 1982], target position (Fu et al., 1995], or the goal of a movement [Alexander and Crutcher, 1990]. Several studies also indicate that M1 neurons can hold premotor information for short periods, which suggests that M1 neurons might exhibit the functional equivalent of elementary memory functions [Alexander and Crutcher 1990; Ashe et al., 1993; Georgopoulos et al., 1989; Kalaska and Crammond, 1992]. The present data demonstrate that patterns of BOLD activation in M1 differentiate between types of imagined contralateral hand actions and thus underpin the importance of M1 for cognitive functions within and for the motor system. This fits with the idea that M1 represents higher order movement variables, and suggests that this role might emerge from distributed networks within M1 rather than discrete representations.

Regarding the posterior parietal subareas, we found several regions of the PPC to carry information regarding the type of imagined action. However, the best decoding performance was found within the superior parietal lobe of both hemispheres. Interestingly, it has been argued that neural signals in the PPC as well as the dorsal section of the PMC are related to motor intentions and the final goal rather than to the single steps of a movement [Andersen and Cui, 2009; Desmurget et al., 2009; Hoshi and Tanji, 2007]. Against this background, it is tempting to speculate that goal representations may have played a crucial role for the decoding of the different action types we observed. Future studies could test the relative importance of movement sequences and action intentions for representations in the PPC and other areas by independently varying these factors. This would reveal whether, for example, action intentions can be cross‐classified across different types of imagined movement sequences to achieve the same goal or vice versa.

In an applied context, the current results might point to relevant target areas for up‐coming techniques such as neural prosthetic applications. Our results suggest that it might be possible to decode action‐specific information for MI from frontal as well as parietal areas, and this could be helpful in cases of localized brain damage due to stroke [see also Filimon et al., 2014; Gallivan et al., 2011b, 2013]. In this regard, an interesting study by Aflalo et al. [2015] demonstrated that MI of movements with different goals and trajectories could be decoded from neural populations within the human PPC of a tetraplegic subject. These and our results suggest that the PPC/SPL or the dorsal section of the PMC might be especially promising candidate areas for such applications as they might represent high‐level aspects of action [Fogassi and Luppino, 2005]. In this context, it would also be useful to learn more about the specific roles of single areas in the motor hierarchy. As mentioned above, different dimensions of actions and different aspects of the motor process (the action goal or movement characteristics, such as speed, accuracy, and effort) might be represented by different areas. Future studies could test this by varying these factors parametrically and independently of each other.

## CONCLUSION

The present study investigated whether MI of different hand actions is accompanied by action‐specific patterns of activation in areas of the human core and broader motor regions. Our findings show that decoding MI content is possible for the left premotor region (especially its dorsal section), the posterior parietal region of both hemispheres, left intraparietal sulcus, right inferior parietal lobe, the superior parietal lobe of both hemispheres, and left M1 as well as visual areas. Moreover, control analyses showed that accurate decoding of action types did not hinge on differences in mean amplitude levels. These data demonstrate that activations within the frontal and posterior parietal motor regions carry information regarding the *content* of motor imagery, that is, the type of imagined action. Thus, they appear to be the likely locus for the representation of MI content in the human brain. Future studies should investigate how the representation of different dimensions of MI is distributed across these areas.

## Supporting information

Supporting Information
