# Supplementary material for: Motor imagery of hand actions: Decoding the content of motor imagery from brain activity in frontal and parietal motor areas
Source: Hum Brain Mapp. 2015 Oct 9;37(1):81–93. doi: 10.1002/hbm.23015 (PMC4737127; doi:10.1002/hbm.23015)
Supplement: Supplementary file 1 — Supporting Information [file HBM-37-81-s001.docx]

Supplementary Material

Univariate Analysis:

In addition to mean amplitude levels across ROIs (Figure 2 c), we investigated univariate effects in a “traditional” mass-univariate analysis. Calculating the contrasts for the different MI conditions against the rest condition, we found several activation sites that captured broad areas within the premotor as well as within the posterior parietal section. More precisely, we found activation for imagined aiming movements compared to rest capturing the left dorsal premotor area as well as the adjoining pre-SMA, the SMA proper, the SPL, and the IPL of the left hemisphere. For motor imagery of extension–ﬂexion movements, we found activation clusters capturing the left pre-SMA and SMA proper as well as the ventral section of the premotor area of the left hemisphere. For posterior parietal sections, we found no activation cluster passing the threshold. Furthermore, we found activation clusters for imagined squeezing movements within the dorsal and ventral section of the PMC of the left hemisphere, the pre-SMA, the SMA proper, as well as within the left IPL (Area PF) and the posterior section of the left SPL (Area 7P) (Fig. S1).

To detect whether the different hand movements are mapped action-speciﬁcally within the premotor area and the posterior parietal area, we contrasted the different action conditions to deﬁne action-speciﬁc sections within our ROIs. Calculating the respective contrasts (Imagery Squeezing vs. [Imagery Aiming + Imagery Extension–Flexion]; Imagery Aiming vs. [Imagery Squeezing + Imagery Extension–Flexion]; Imagery Extension–Flexion vs. [Imagery Squeezing + Imagery Aiming]) revealed clearly deﬁned action-speciﬁc sections associated speciﬁcally with squeezing movements in an activation cluster that captured area 7a of the superior posterior parietal lobe of the left hemisphere. Furthermore, activation sites specific for imagined squeezing movements were detected within the SMA proper of the right hemisphere. We found action-specific activation associated with extension**–**flexion movements in the parietal operculum of the right hemisphere. We did not ﬁnd any activation sites within the predefined ROIs for aiming movements that passed the statistical threshold (Fig S1). All results and the speciﬁc coordinates of the single activation clusters are summarized in Table S1.


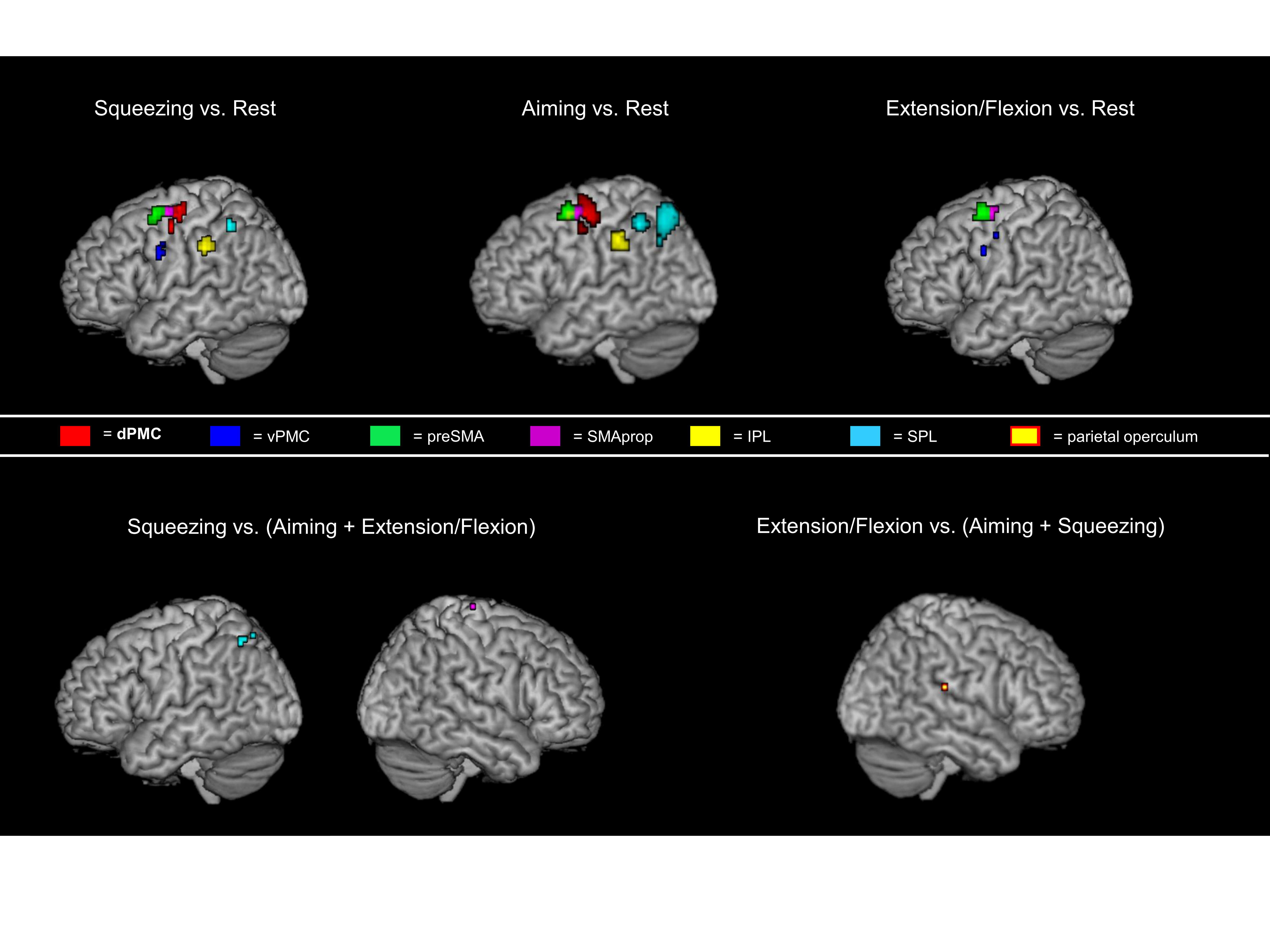
**Figure S1.** Results of the univariate ROI analysis. Colors indicate significantly activated clusters within the respective regions of interest as follows: red: dorsal premotor cortex, blue: ventral premotor cortex, green: preSMA, violet: SMAproper, yellow: IPL, turquoise: SPL, yellow with red frame: parietal operculum.

**Table S1** Results of the univariate ROI analysis

|  | Left/Right | Cluster size | MNI coordinates of max. *t* value | | | *max. t* value |
| --- | --- | --- | --- | --- | --- | --- |
|  |  |  | *X* | *y* | *z* |  |
| *Aiming vs. Rest* |  |  |  |  |  |  |
| IPL (PF) | L | 68 | -51 | -34 | 43 | 4.17 |
| SPL (7a) | L | 74 | -24 | -67 | 55 | 5.25 |
| SPL (7PC) | L | 14 | -12 | -73 | 55 | 3.63 |
| SPL (7P) | L | 8 | -36 | -49 | 55 | 4.87 |
| SMA(prop) | L | 47 | -6 | -4 | 58 | 4.82 |
| dPMC | L | 280 | -21 | -10 | 58 | 6.88 |
| preSMA | L | 56 | -6 | 5 | 58 | 4.86 |
|  |  |  |  |  |  |  |
| *Extension–Flexion vs. Rest* | | |  |  |  |  |
| SMA(prop) | L | 45 | -6 | -4 | 58 | 483 |
| preSMA | L | 61 | -6 | 5 | 58 | 5.49 |
| vPMC | L | 42 | -57 | 2 | 34 | 3.87 |
|  |  |  |  |  |  |  |
| *Squeezing vs. Rest* | | |  |  |  | |
| IPL (PF) | L | 66 | -57 | -31 | 37 | 4.92 |
| SPL (7p) | L | 6 | -36 | -49 | 55 | 4.1 |
| SMA(prop) | L | 60 | -6 | -4 | 58 | 4.34 |
| dPMC | L | 266 | -42 | -7 | 52 | 4.96 |
| preSMA | L | 61 | -6 | 5 | 58 | 5.2 |
| vPMC | L | 46 | -57 | 2 | 34 | 4.34 |
|  |  |  |  |  |  |  |
| *Squeezing vs. (Extension–Flexion+Aiming)* | | |  |  |  | |
| SPL (7a) | L | 104 | -21 | -61 | 52 | 4.34 |
| SMA(prop) | R | 11 | 9 | -22 | 76 | 4.43 |
|  |  |  |  |  |  |  |
| *Extension–Flexion vs. (Aiming+Squeezing)* | | |  |  |  | |
| Parietal Operculum | R | 6 | 42 | -28 | 19 | 3.86 |
| *P* < 0.05,, FWE-corrected in ROIs. For abbreviations, see text. | | | |  |  |  |

ROI sizes

**Table S2**: Sizes of the Region of Interest

|  | | | | | |
| --- | --- | --- | --- | --- | --- |
|  | N | Minimum | Maximum | Mean | SD |
| M1_r | 20 | 129,00 | 254,00 | 180,5000 | 28,29450 |
| M1_l | 20 | 138,00 | 232,00 | 182,3000 | 28,95205 |
| dPMC_r | 20 | 48,00 | 108,00 | 81,2000 | 16,77906 |
| dPMC_l | 20 | 39,00 | 129,00 | 75,8500 | 19,96649 |
| vPMC_r | 20 | 68,00 | 140,00 | 101,5000 | 22,72837 |
| vPMC_l | 20 | 73,00 | 127,00 | 95,5000 | 15,88279 |
| IPS_r | 20 | 122,00 | 255,00 | 197,7000 | 36,48374 |
| IPS_l | 20 | 159,00 | 227,00 | 195,4000 | 19,24195 |
| SPL_r | 20 | 122,00 | 208,00 | 159,9500 | 25,70577 |
| SPL_l | 20 | 135,00 | 238,00 | 187,8000 | 25,71422 |
| IPL_r | 20 | 128,00 | 229,00 | 180,0000 | 32,26045 |
| IPL_l | 20 | 136,00 | 249,00 | 189,5500 | 28,99269 |
| Premotor_r | 20 | 118,00 | 234,00 | 182,7000 | 29,05367 |
| Premotor_l | 20 | 114,00 | 240,00 | 171,3500 | 30,13527 |
| Parietal_l | 20 | 444,00 | 671,00 | 572,7500 | 58,42573 |
| Parietal_r | 20 | 382,00 | 673,00 | 537,6500 | 81,42951 |
|  |  |  |  |  |  |
